# Effect of selective serotonin reuptake inhibitors use on endocrine therapy adherence and breast cancer mortality: a population-based study

**DOI:** 10.1007/s10549-016-3928-3

**Published:** 2016-08-05

**Authors:** Antonis Valachis, Hans Garmo, John Weinman, Irma Fredriksson, Johan Ahlgren, Malin Sund, Lars Holmberg

**Affiliations:** 1 Centre for Clinical Research Sörmland, Uppsala University, 63188 Eskilstuna, Sweden; 2 Division of Cancer Studies, Cancer Epidemiology Unit, School of Medicine, King’s College London, London, UK; 3 Institute of Pharmaceutical Science, King’s College London, London, SE1 9NH UK; 4 Department of Molecular Medicine and Surgery, Karolinska Institutet, Karolinska University Hospital, Stockholm, Sweden; 5 Department of Oncology, Faculty of Medicine and Health, University of Örebro, Örebro, Sweden; 6 Department of Surgical and Perioperative Sciences, Umeå University, 901 85 Umeå, Sweden

**Keywords:** Breast cancer, SSRI, Adherence, Endocrine therapy, Prognosis, Tamoxifen

## Abstract

The purpose of the study was to investigate whether the concomitant use of selective serotonin reuptake inhibitors (SSRI) with tamoxifen influences the risk of death due to breast cancer, and we also investigated the association between SSRI use and adherence to oral endocrine therapy (ET). We analyzed data from BCBaSe Sweden, which is a database created by the data linkage of Registries from three different regions of Sweden. To investigate the association between ET adherence and SSRI use, we included all women who were diagnosed with non-distant metastatic ER-positive invasive breast cancer from July 2007 to July 2011 and had at least one dispensed prescription of oral tamoxifen or aromatase inhibitor. To investigate the role of concurrent administration of SSRI and tamoxifen on breast cancer prognosis, we performed a nested case–control study. In the adherence cohort, 9104 women were included in the analyses. Women who received SSRI, either before or after breast cancer diagnosis, were at higher risk for low adherence to ET. However, when the overlapping period between SSRI use and ET was >50 %, no excess risk for low adherence was observed. Non-adherence (<80 %) to ET was significantly associated with worse breast cancer survival (OR 4.07; 95 % CI 3.27–5.06). In the case–control study, 445 cases and 11125 controls were included. The concomitant administration of SSRI and tamoxifen did not influence breast cancer survival, neither in short-term (OR 1.41; 95 % CI 0.74–2.68) nor in long-term SSRI users (OR 0.85; 95 % CI 0.35–2.08). Concomitant SSRI and tamoxifen use does not seem to increase risk for death due to breast cancer. Given the positive association between continuing antidepressive pharmacotherapy for a longer period of time and adherence to ET, it is essential to capture and treat depression in breast cancer patients to secure adherence to ET.

## Introduction

In patients with estrogen receptor (ER)-positive breast cancer, adjuvant endocrine therapy, such as tamoxifen and aromatase inhibitors (AIs), reduces the risk of breast cancer recurrence and death [1, 2]. However, a considerable proportion of patients discontinue their oral endocrine treatment [3] despite the risk of jeopardizing their chances of survival from breast cancer [4, 5]. There are many reasons why people do not adhere to medical treatments [6] and a significant one of these is depression [7], which is fairly prevalent in patients with breast cancer [8]. There is some evidence that the concomitant use of antidepressant and endocrine therapy in breast cancer is associated with a poorer clinical outcome but the reasons for this have not been fully explored [9, 10]. Two distinct, but not mutually exclusive, explanations are possible for this, namely an adherence effect and a drug interaction effect [4, 9].

Depression reduces the adherence to oral endocrine therapy [11]. The association between depression, antidepressant therapy, and adherence to endocrine therapy is complex, and several aspects need further investigation. First, the role of antidepressant therapy before initiation of endocrine therapy on adherence is unclear. Barron et al. reported an association between antidepressant use before breast cancer diagnosis and non-persistence to tamoxifen therapy [12], but He et al. could not reveal a similar association in a larger dataset [13]. Second, whether continuing antidepressant therapy after breast cancer diagnosis affects adherence to endocrine therapy has not been studied. In addition, the effect of time to antidepressants initiation in respect to breast cancer diagnosis on adherence has not been studied either.

A second explanation of the negative effect of the concomitant use of antidepressants and oral endocrine therapy is the potential risk for reduced activity of endocrine therapy due to drug interaction. The biologic rationale for this is that tamoxifen is a pro-drug that is catalyzed to its active substance endoxifen predominantly by cytochrome P450 isoenzyme 2D6 (CYP2D6) [14]; a cytochrome that can be inhibited by the selective serotonin reuptake inhibitors (SSRIs) antidepressants. If this interaction has a clinically relevant effect, the consequences for clinical management are substantial: Tamoxifen has key role as adjuvant endocrine therapy in a large proportion of women with early breast cancer [1]; a considerable proportion of breast cancer patients suffer from significant depressive symptoms [8]; the current recommendation is to use SSRIs as first-line treatment against depression [15, 16].

Several studies have tried to investigate the clinical effect of this potential interaction but have been hampered by a limited number of events [17, 18], the use of a too low cut-off for acceptable adherence rate [17], and the lack of analyses on the effect of the length of time with concomitant use of tamoxifen and SSRI [4, 19]. Two studies could overcome these limitations, but their results are contradictory. Kelly et al. showed an association between a specific SSRI medication, paroxetine, and increased risk of death from breast cancer [10]. However, Haque et al. could not find an increased risk for breast cancer recurrence in patients who concurrently used tamoxifen and SSRI, including paroxetine [20]. As a result, whether a clinically significant interaction between SSRI and tamoxifen as it is supported by preclinical data exists is still controversial.

We investigated whether the concomitant use of SSRI and tamoxifen influences the risk of death due to breast cancer. Since any such impact could be due to either drug interaction or pattern of adherence, our secondary purpose was to investigate the association between SSRI use, in both the prediagnostic and the postdiagnostic period of breast cancer, and adherence to oral endocrine therapy.

## Patients and methods

### Data source

The current study was based on data linkage of The Regional Breast Cancer Clinical Quality Registers of the Uppsala/Örebro, Stockholm-Gotland, and Northern regions of Sweden. These registries represent approximately 60 % of breast cancer patients in Sweden and contain information on date of diagnosis, tumor characteristics, primary treatment, and follow-up on all newly diagnosed breast cancer patients with less than 5 % missing information, in terms of diagnosis characteristics, of patients when validated against the National Swedish Cancer Register.

The Swedish Prescribed Drug Register contains information on all the prescribed medications dispensed in Swedish pharmacies since July 1, 2005. The database provides information on each dispensed drug including dates of dispensation, number of defined daily doses (DDD), and classification of the drugs based on the anatomic therapeutic chemical (ATC) system.

Individual-level information on socioeconomic and demographic factors was obtained from the Longitudinal integration database for health insurance and labor market studies (LISA) managed by Statistics Sweden. This nationwide database integrates existing data from labor market-, educational-, and social-sector registers and contains data from 1990 and onward on all individuals 16 years or older residing in Sweden and is updated yearly. Finally, the Swedish Inpatient Register contains information on hospital admission dates and diagnosis of diseases and has had complete national coverage since 1987.

Information from the above registers was linked using a ten-digit personal identifier numbers assigned for all persons registered in Sweden.

### Study population

We identified all women residing in the Uppsala/Örebro, Stockholm-Gotland, and Northern healthcare regions of Sweden who were diagnosed with ER-positive invasive breast cancer without distant metastasis between January 1, 2004 and July 31, 2011 and had at least one dispensed prescription of tamoxifen (ATC code: L02BA01) or AI (anastrozole, exemetastane, or letrozole; ATC code: L02BG) after diagnosis. We included only patients who were alive for at least 2.5 years after breast cancer diagnosis because of the risk to underestimate the adherence rate in patients with early disease progression. In those patients, the endocrine therapy has probably been stopped by the treating physician, causing a falsely low adherence rate. This patient subgroup is not interesting to be included in a study investigating a potential drug interaction effect because the breast cancer recurrence is most probably related to an aggressive biology of the tumor.

### Cohort for adherence study

For the adherence part of our study, we included patients from our study population who were diagnosed with breast cancer from 1/7/2007. Using this time period, we were able to study the role of prediagnostic SSRI use on adherence for a maximum of 2 years before diagnosis since the Swedish Prescribed Drug Register was started 1/7/2005.

### Nested case–control study

To investigate the role of concurrent administration of SSRI and tamoxifen on breast cancer prognosis, we created a nested case–control study. As cases we defined all patients in our study population who died due to breast cancer until the last date of follow-up (31/12/2014). Control patients (25 controls for each case) were selected using the incidence density sampling after stratification for region and year for diagnosis.

### Adherence to oral endocrine treatment

Adherence to oral endocrine therapy was investigated during the first 2 years after breast cancer diagnosis after a run-in period of 6 months to minimize the risk for overestimation of non-adherence due to patients that were treated with chemotherapy after breast cancer diagnosis.

Patterns of adherence were assessed based on tamoxifen and AI-dispensed prescriptions recorded in the Swedish Prescribed Drug Register. Adherence to oral endocrine treatment was measured by calculating the medication possession ratio (MPR) [21, 22]. MPR is defined as number of days of dispensed prescribed supplies divided by the number of days in study period × 100 %. Actual prescribed daily dose (PDD) for each patient was used for the MPR calculations.

### Exposure to SSRI

Any prescription of SSRI during the study follow-up period was identified, irrespective of the type of SSRI. However, we excluded patients with serotonin and norepinephrine reuptake inhibitor (SNRI) prescription to avoid inclusion of patients treated with SNRI due to endocrine therapy-related vasomotor symptoms (the SNRI venlafaxin is the only antidepressant that is recommended against vasomotor symptoms by the National Swedish breast cancer guidelines [23]) rather than depression.

To investigate the effect of SSRI exposure on both adherence and breast cancer survival, we divided the follow-up time after breast cancer diagnosis into 3-month periods. We then calculated the use of SSRI in each of these 3-month periods.

The coverage period with SSRI use was <25 % if SSRI was used in 0–1 of the 3-month periods; 25–50 % if SSRI was used in 2–3 of the 3-month periods; 50–75 % if SSRI was used in 4–5 of the 3-month periods; >75 % if SSRI was used in 6–8 of the 3-month periods.

For the nested case–control part of the study, we also divided the follow-up time into 3-month periods. We defined as short-term SSRI users those patients that received SSRI for 1–6 of the 3-month periods whereas long-term SSRI users were defined as those who received SSRI for ≥7 of the 3-month periods.

### Endpoints

The endpoint in the nested case–control study was the odds ratio (OR) of breast cancer death in relation to SSRI exposure during study follow-up.

For the adherence cohort study, the endpoint was the rate of adherence to oral endocrine therapy. We used a cut-off value of 80 % to define patients with high versus low adherence. A patient with MPR <80 % was defined as patient with low adherence to oral endocrine therapy.

### Covariates

Tumor-related characteristics (year of breast cancer diagnosis, tumor size, lymph node status, tumor grade, and progesterone-receptor status), treatment-related factors (type of primary surgery, chemotherapy, and radiation therapy), and patient-related factors (age at diagnosis) were obtained from the Regional Quality Registers. Comorbidities were assessed by calculating the Charlson comorbidity index (CCI) based on information on hospital admissions for diseases other than breast cancer recorded in the Swedish Inpatient Register since 10 years prior to the start of follow-up. From the LISA database, we obtained information on socioeconomic factors including education level.

### Statistical analysis

Univariate and multivariable conditional logistic regression were used to estimate OR and their accompanying 95 % confidence intervals (CI) of breast cancer death in the case–control study. ORs in the multivariable models were adjusted for age, CCI, stage, grade, progesterone-receptor status, type of surgery, radiotherapy, and chemotherapy.

Univariate and multivariable logistic regression was use to estimate OR with associated 95 % CIs of the dichotomized outcome MPR <80 % (dependent variable) [24]. The multivariable models were adjusted for the same set of covariates as in the conditional logistic regression above.

## Results

We identified 18,432 women with ER-positive breast cancer without distant metastasis treated with adjuvant endocrine therapy during the study period (Jan 1, 2004–July 31, 2011). In total, 445 women died due to breast cancer by the end of follow-up.

**Fig. 1:**
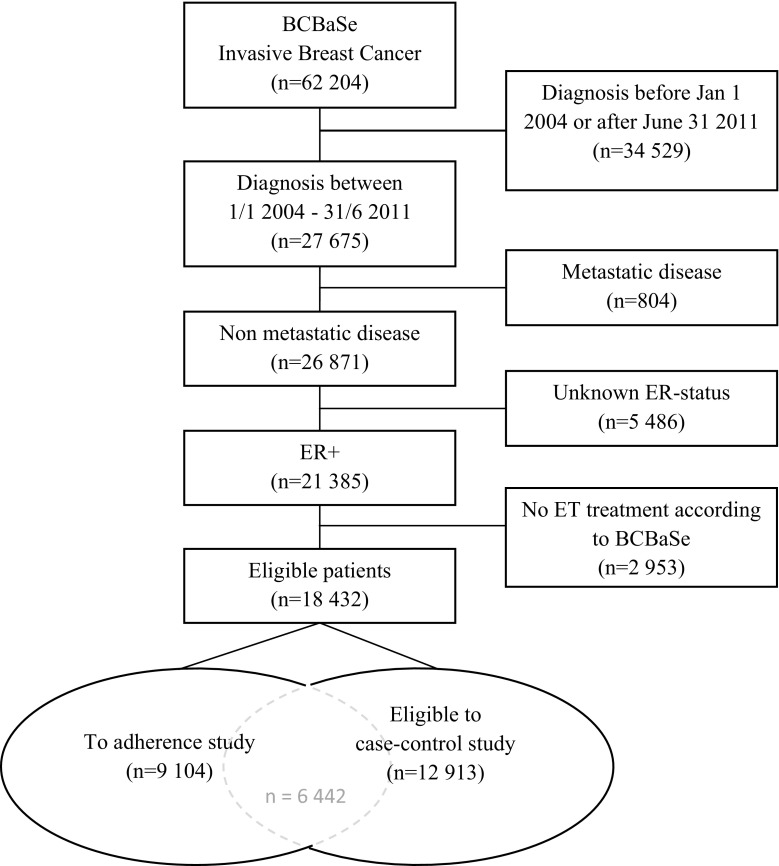
Flow chart of study participants. *ER* Estrogen receptor, *ET* endocrine therapy

The nested case–control study included 445 cases (deaths due to breast cancer) and 11125 controls (Fig. 1).The adherence cohort included 9104 women who were diagnosed with non-metastatic ER-positive breast cancer from July 2007 to July 31, 2011.

**Fig. 2:**
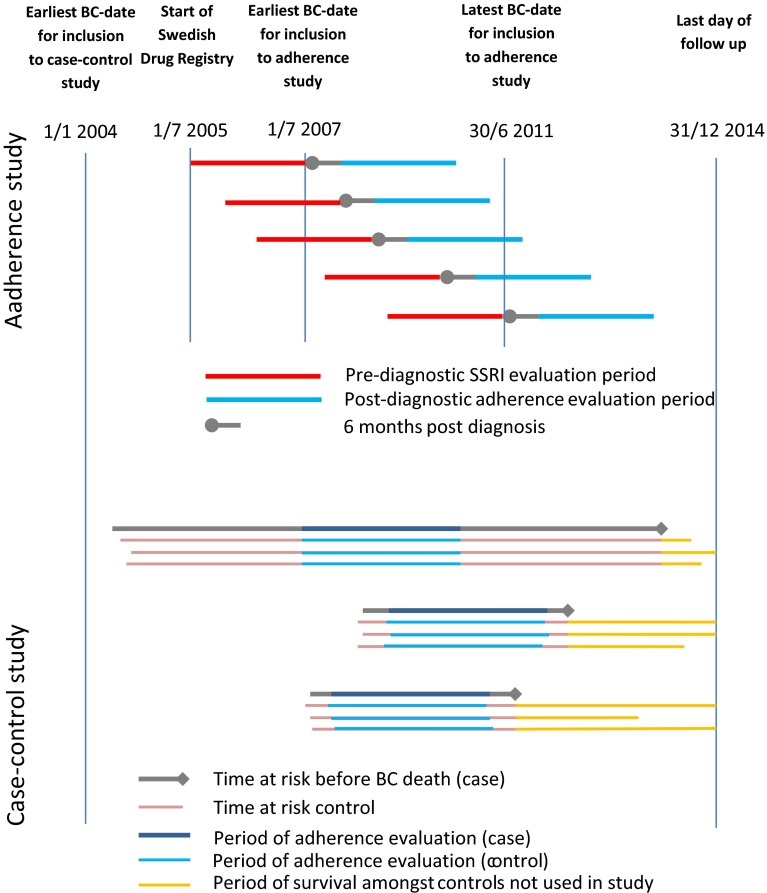
Description of study design. *SSRI* Selective serotonine receptor inhibitor, *BC* breast cancer

The study design with the different patient cohorts are outlined in Fig. 2.

### SSRI use and adherence

Of 9104 women in the adherence cohort, 731 (8 %) used SSRI. Of those, 36 % had no history of SSRI use before breast cancer diagnosis. The characteristics of the participants included in the adherence cohort are presented in Table 1.

**Table 1:** Characteristics of participants included in adherence cohort

	No SSRI prior to BC diagnosis (*n* = 8373)	SSRI prior to BC diagnosis (*n* = 731)	All (*n* = 9104)
Year of cancer diagnosis, *n* (%)			
2007–2009	4936 (59.0)	448 (61.3)	5384 (59.1)
2010–June 2011	3437 (41.0)	283 (38.7)	3720 (40.9)
Age at cancer diagnosis, *n* (%)			
<40	303 (3.6)	31 (4.2)	334 (3.7)
40–49	1282 (15.3)	148 (20.2)	1430 (15.7)
50–59	1811 (21.6)	194 (26.5)	2005 (22.0)
60–69	2750 (32.8)	220 (30.1)	2970 (32.6)
70–79	1443 (17.2)	90 (12.3)	1533 (16.8)
80–89	723 (8.6)	47 (6.4)	770 (8.5)
90+	61 (0.7)	1 (0.1)	62 (0.7)
Tumor size, *n* (%)			
≤20 mm	5346 (63.8)	482 (65.9)	5828 (64.0)
20.1–50 mm	2648 (31.6)	217 (29.7)	2865 (31.5)
>50 mm	329 (3.9)	29 (4.0)	358 (3.9)
Missing data	50 (0.6)	3 (0.4)	53 (0.6)
N-stage, *n* (%)			
N0	7286 (87.0)	639 (87.4)	7925 (87.0)
N+	1054 (12.6)	87 (11.9)	1141 (12.5)
NX/missing	33 (0.4)	5 (0.7)	38 (0.4)
M-stage, *n* (%)			
M0	7286 (87.0)	630 (86.2)	7916 (87.0)
MX/missing	1087 (13.0)	101 (13.8)	1188 (13.0)
Grade, *n* (%)			
Well	1824 (21.8)	171 (23.4)	1995 (21.9)
Moderately	4616 (55.1)	399 (54.6)	5015 (55.1)
Poorly	1747 (20.9)	147 (20.1)	1894 (20.8)
Missing	186 (2.2)	14 (1.9)	200 (2.2)
Treatment, *n* (%)			
Breast conserving surgery	4888 (58.4)	420 (57.5)	5308 (58.3)
Mastectomy	3440 (41.1)	305 (41.7)	3745 (41.1)
Other surgery	25 (0.3)	4 (0.5)	29 (0.3)
Chemotherapy	2892 (34.5)	278 (38.0)	3170 (34.8)
Radiotherapy	6179 (73.8)	559 (76.5)	6738 (74.0)
CCI, *n* (%)			
0	7278 (86.9)	623 (85.2)	7901 (86.8)
1	574 (6.9)	54 (7.4)	628 (6.9)
2	370 (4.4)	37 (5.1)	407 (4.5)
3+	151 (1.8)	17 (2.3)	168 (1.8)
Educational level, *n* (%)			
High	2915 (34.8)	273 (37.3)	3188 (35.0)
Middle	3347 (40.0)	291 (39.8)	3638 (40.0)
Low	2025 (24.2)	160 (21.9)	2185 (24.0)
Missing	86 (1.0)	7 (1.0)	93 (1.0)

*BC* Breast cancer, *CCI* Charlson comorbidity index

SSRI use was associated with higher risk for low adherence to oral endocrine therapy and this association was significant in both patients who received SSRI prior to breast cancer diagnosis (OR 1.33; 95 % CI 1.03–1.73) and those with SSRI during the first months after breast cancer diagnosis (OR 1.37; 95 % CI 1.01–1.85) (Table 2).

**Table 2:** Association between non-adherence to endocrine therapy and SSRI use

	Cases with adherence <0.8	%	Crude model		Multivariate model	
			OR	95 % CI	OR	95 % CI
SSRI usage prior to BC diagnosis						
No SSRI	1307	15.0	1.00	Ref.	1.00	Ref.
SSRI	75	18.9	1.32	1.02–1.71	1.33	1.03–1.73
SSRI during 6-month period from BC diagnosis						
No SSRI	1327	15.1	1.00	Ref.	1.00	Ref.
SSRI	55	19.0	1.33	0.98–1.79	1.37	1.01–1.85
SSRI during 6-month period from BC diagnosis without SSRI prior to BC diagnosis						
No SSRI	1373	15.1	1.00	Ref.	1.00	Ref.
SSRI	9	23.7	1.74	0.82–3.68	1.76	0.83–3.76
SSRI usage prior to BC diagnosis but not during 6-month period after BC diagnosis						
No SSRI	1353	15.1	1.00	Ref.	1.00	Ref.
SSRI	29	19.9	1.39	0.92–2.10	1.34	0.89–2.03
SSRI prior to 6 month from BC diagnosis						
No SSRI	1298	15.0	1.00	Ref.	1.00	Ref.
SSRI	84	19.3	1.36	1.06–1.74	1.37	1.07–1.76
SSRI during month 7-31 from BC diagnosis						
No SSRI	1233	14.7	1.00	Ref.	1.00	Ref.
SSRI <25 % of time	59	24.7	1.90	1.41–2.56	1.96	1.45–2.65
SSRI 25–50 % of time	27	20.5	1.49	0.97–2.28	1.48	0.97–2.28
SSRI 50–75 % of time	23	18.3	1.29	0.82–2.04	1.32	0.83–2.09
SSRI ≥0.75 % of time	40	17.1	1.19	0.85–1.69	1.17	0.83–1.66
SSRI during month 7-31 from BC diagnosis and history of SSRI						
No SSRI	1233	14.7	1.00	Ref.	1.00	Ref.
SSRI <25 % of time, no usage prior to month 7	27	25.5	1.98	1.27–3.08	2.06	1.32–3.22
SSRI 25–50 % of time, no usage prior to month 7	19	22.1	1.64	0.98–2.74	1.66	0.99–2.78
SSRI 50–75 % of time , no usage prior to month 7	14	23.3	1.76	0.97–3.22	1.78	0.97–3.26
SSRI ≥75% of time, no usage prior to month 7	5	11.4	0.74	0.29–1.89	0.67	0.26–1.71
SSRI <50 % of time and usage prior to month 7	40	22.3	1.67	1.17–2.38	1.69	1.18–2.41
SSRI ≥50 % of time and usage prior to month 7	44	17.2	1.20	0.86–1.67	1.21	0.87–1.69

*RR* Relative risk, *CI* confidence interval, *BC* breast cancer

When we analyzed the association between SSRI use and adherence based on the proportion of time on endocrine therapy that overlapped with SSRI use, we found that there was a higher risk for low adherence in patients when the overlapping period was <50 %, whereas in patients with >50 % overlapping period this statistically significant association disappeared (Table 2).

Considering the effect of non-adherence on breast cancer prognosis, we found that the dichotomized adherence with an 80 % cut-off was significantly associated with worse breast cancer survival when adjusted for other prognostic factors (OR 4.07; 95 % CI 3.27–5.06).

### Association between SSRI use during endocrine treatment and prognosis

The characteristics of 445 cases and 11125 controls included in the case–control study are presented in Table 3.

**Table 3:** Characteristics of participants included in case–control study

	Case (*n* = 445)	Control (*n* = 11 125)	All (*n* = 11 570)
Year of cancer diagnosis, *n* (%)			
2004–2005	201 (45.2)	5025 (45.2)	5226 (45.2)
2006–2007	159 (35.7)	3975 (35.7)	4134 (35.7)
2008–2010	85 (19.1)	2125 (19.1)	2210 (19.1)
Age at cancer diagnosis, *n* (%)			
<40	33 (7.4)	405 (3.6)	438 (3.8)
40–49	60 (13.5)	1851 (16.6)	1911 (16.5)
50–59	86 (19.3)	2860 (25.7)	2946 (25.5)
60–69	116 (26.1)	3508 (31.5)	3624 (31.3)
70–79	88 (19.8)	1824 (16.4)	1912 (16.5)
80–89	56 (12.6)	645 (5.8)	701 (6.1)
90+	6 (1.3)	32 (0.3)	38 (0.3)
TNM-stage, *n* (%)			
I	91 (20.4)	6132 (55.1)	6223 (53.8)
II	264 (59.3)	4129 (37.1)	4393 (38.0)
III	52 (11.7)	243 (2.2)	295 (2.5)
IV	20 (4.5)	375 (3.4)	395 (3.4)
Missing data	18 (4.0)	246 (2.2)	264 (2.3)
PR-status, *n* (%)			
PR−	134 (30.1)	2082 (18.7)	2216 (19.2)
PR+	302 (67.9)	8928 (80.3)	9230 (79.8)
Missing data	9 (2.0)	115 (1.0)	124 (1.1)
Grade, *n* (%)			
1	26 (5.8)	2384 (21.4)	2410 (20.8)
2	228 (51.2)	6242 (56.1)	6470 (55.9)
3	161 (36.2)	2081 (18.7)	2242 (19.4)
Missing data	30 (6.7)	418 (3.8)	448 (3.9)
Treatment, *n* (%)			
Local surgery	154 (34.6)	6637 (59.7)	6791 (58.7)
Mastectomy	277 (62.2)	4306 (38.7)	4583 (39.6)
No surgery/missing data	14 (3.1)	182 (1.6)	196 (1.7)
Chemotherapy	211 (47.4)	3305 (29.7)	3516 (30.4)
Radio therapy	310 (69.7)	8016 (72.1)	8326 (72.0)
CCI, *n* (%)			
0	382 (85.8)	9925 (89.2)	10307 (89.1)
1	33 (7.4)	676 (6.1)	709 (6.1)
2	19 (4.3)	378 (3.4)	397 (3.4)
3+	11 (2.5)	146 (1.3)	157 (1.4)
Educational level, *n* (%)			
High	124 (27.9)	3852 (34.6)	3976 (34.4)
Middle	174 (39.1)	4394 (39.5)	4568 (39.5)
Low	133 (29.9)	2765 (24.9)	2898 (25.0)
Missing data	14 (3.1)	114 (1.0)	128 (1.1)

*PR* Progesterone-receptor, *CCI* Charlson comorbidity index

In the primary analysis including all the patients treated with tamoxifen therapy irrespective of the adherence level, patients with concomitant short-term SSRI use had worse breast cancer survival than non-users (adjusted OR 1.58; 95 % CI 1.00–2.49), whereas no difference was observed between long-term SSRI users and non-users (adjusted OR 0.68; 95 % CI 0.29–1.57) (Table 4).

**Table 4:** Odds ratios for breast cancer death by exposure of SSRI

	Crude model		Multivariate model	
Exposure	OR	95% CI	OR	95% CI
Overall study population				
No SSRI	1.00	Ref.	1.00	Ref.
Short-term SSRI users	1.25	0.81–1.94	1.58	1.00–2.49
Long-term SSRI users	0.58	0.26–1.31	0.68	0.29–1.57
Women with adherence >80%				
No SSRI	1.00	Ref.	1.00	Ref.
Short-term SSRI users	1.18	0.64–2.20	1.41	0.74–2.68
Long-term SSRI users	0.79	0.35–1.81	0.85	0.35–2.08

To study a potential drug interaction, we restricted the study population to women treated with tamoxifen and ≥80 % adherence: neither short-term (OR 1.41; 95 % CI 0.74–2.68) nor long-term SSRI (OR 0.85; 95 % CI 0.35–2.08) use was associated with breast cancer survival (Table 4). When the same analysis was performed restricted to patients that received strong CYP2D6-inhibiting SSRIs (paroxetine, fluoxetine, and fluvoxamine), no statistically significant association between SSRI use and breast cancer survival was revealed (short-term SSRI use, OR 2.00; 95 % CI 0.60–6.71; long-term SSRI use, OR 0.79; 95 % CI 0.18–3.44).

## Discussion

In our population-based study, we found no association between concomitant use of SSRI and tamoxifen and breast cancer-specific survival. The lack of association was consistent even when the analysis was restricted to patients that used strong CYP2D6-inhibiting SSRIs or patients with long-term co-administration of tamoxifen with SSRI. However, poor adherence on endocrine therapy was clearly associated with worse prognosis. Regarding the role of SSRI on adherence to endocrine therapy, we found a negative effect of both pre- and postdiagnostic SSRI use on adherence with the exception of patients with SSRI use for a longer period of time during study follow-up in whom the adherence was comparable to those who have not been treated with SSRI.

Early pharmacological studies revealed a decreased endoxifen plasma concentration in patients who were poor CYP2D6 metabolizers or those who received concomitant CYP2D6 inhibitors, such as SSRIs [9, 25, 26]. This observation raised the hypothesis that the concomitant administration of tamoxifen and SSRIs could have a detrimental effect on tamoxifen efficacy. However, the results from two well-designed, large studies aimed to investigate this hypothesis are contradictory [10, 20]. Kelly et al. found that concomitant use of tamoxifen and paroxetine increased the risk for death due to breast cancer. However, the study did not include data on breast cancer stage which could influence the results by including more patients with late-stage disease in the paroxetine group. On the other hand, Haque et al. could not find any association between SSRI use during tamoxifen and breast cancer recurrence, but no data on breast cancer survival were presented.

Our results that poor adherence to endocrine therapy was associated with worse prognosis are consistent with prior studies [4, 5, 27] and further support the need to develop interventions to improve adherence to endocrine adjuvant therapy.

SSRI users had a worse adherence rate than non-SSRI users. This observation was consistent irrespective of the time of SSRI initiation (before or after breast cancer diagnosis) indicating an association between SSRI use with non-adherence to endocrine therapy. Considering the fact that nearly 80 % of patients with SSRI prescription in Sweden is due to depression [28], we hypothesize that there is an association between depression and non-adherence to endocrine therapy. Our results and hypothesis are in accordance with a recent meta-analysis that showed that patients with depressive symptoms had lower adherence to endocrine therapy [11]. However, an association between medical conditions other than depression in which SSRIs are indicated, i.e., panic or anxiety disorders, and non-adherence to endocrine therapy is also possible and further research is needed. Based on the current evidence about the role of depression on endocrine therapy adherence, it has been hypothesized that effective management of depressive symptoms in patients with cancer aids in promoting long-term adherence to endocrine therapy. Although there is some indirect evidence on the positive effect of psychotherapy consultations as part of management against depression on treatment adherence [29], no data exist on the potential effect of pharmacotherapy for depression on adherence. Interestingly, we found an improvement in adherence in patients who received SSRI for a longer period of time during study follow-up (SSRI use more than 50 % of time, namely more than 1 year). This suggests that if the patients with breast cancer and depression receive SSRI treatment as indicated (1–2 years after stabilization of symptoms for the first episode; 5 years to lifetime for subsequent episode [30–32]), the adherence to endocrine therapy will be improved. This is the first study that suggests a positive effect of antidepressive pharmacotherapy on adherence to cancer therapy.

Our study has some limitations. First, prescription refill does not guarantee that the patient has actually consumed the prescribed medication; as a result, there is a risk for underestimation of non-adherence. However, this underestimation is likely to be similar in SSRI users and non-users. Second, we gathered data on comorbidities from inpatient registry and there is a considerably risk that some common comorbidities might be missed. However, the adjustment of our analyses with the CCI measure, which is a measure of more severe comorbid conditions, did not influence the results, indicating that comorbidities do not seem to affect the results. Third, we had no data on the CYP2D6 genotype in our cohort. However, a recent meta-analysis concluded that there is no evidence that different CYP2D6 polymorphisms affect breast cancer prognosis in breast cancer patients treated with tamoxifen [33]. Another potential limitation of our study is the lack of data on the indication of SSRI use in our cohort. Some SSRIs may have been used for the treatment of hot flashes related to endocrine therapy. However, the most evidence-based and commonly used antidepressant for this indication is venlafaxine, which is the only recommended antidepressant against hot flashes by the national swedish breast cancer guidelines [23], and therefore, we excluded this drug from our analyses to minimize the risk of bias. Moreover, venlafaxine is a non-significant inhibitor of CYP2D6 [34]. Furthermore, our study concentrated on SSRIs and did not take into account other antidepressants. The proportion of women receiving antidepressants other than SSRIs is considered low in our cohort because of the recommendation to use SSRI as first-line therapy against depression in Sweden [15]. Finally, our analyses did not take into account the concomitant use of other strong CYP2D6 inhibitors. However, the clinical indication for the use of other strong CYP2D6 inhibitors (bupropion, quinidine) is limited and as result the number of patients treated with these medications is expected to be low. In addition, differences in use of other strong CYP2D6 inhibitors by SSRI usage are not expected in our cohort.

In conclusion, we could not find a negative effect of SSRI use during tamoxifen treatment with respect to risk of breast cancer death even in subgroups of patients that the biological rationale behind the hypothesis was supposed to be stronger (use of strong CYP2D6-inhibitors or use of SSRIs for a long period concomitant with tamoxifen). We suggest that, in breast cancer patients who are successfully treated with SSRIs and are planning to start with tamoxifen, there is no need to change SSRI therapy. However, physicians should discuss other treatment options instead of strong CYP2D6-inhibiting SSRIs with their patients with breast cancer on tamoxifen that need to start with antidepressive therapy, given the biological rationale and the results from one previous well-designed epidemiological study [20]. Finally, depressive symptoms should be captured early in breast cancer patients and adequate pharmacotherapy should be initiated with support to facilitate adherence to both antidepressants and tamoxifen.
